# Treatment for substance use disorders: the Belgian Treatment Demand Indicator registration protocol

**DOI:** 10.1186/s13690-016-0139-7

**Published:** 2016-07-01

**Authors:** Jérôme Antoine, Karin De Ridder, Els Plettinckx, Peter Blanckaert, Lies Gremeaux

**Affiliations:** WIV-ISP: Scientific Institute of Public Health, Brussels, Belgium, Rue Juliette Wytsmanstraat, 14, 1050 Brussels, Belgium

**Keywords:** Substance use disorder, Treatment, Alcohol, Illicit drugs, Registration, Protocol

## Abstract

**Background:**

Registration of patients with substance use disorders is of key importance to get insights and to study trends in patients characteristics and substance use patterns. The Treatment Demand Indicator (TDI) is gathering this information at European level since 2000. In Belgium, this registration started at national level in 2011 and an increasing number of facilities of different types are participating in this data collection since then.

**Methods/Design:**

This surveillance register collects information on every treatment episode started by patients for their substance use disorder. Information is collected on socio-demographic characteristics of the patient, treatment history and substance use patterns. Patients are identified uniquely using their national identification number in order identify multiple episodes followed by a same person. A large range of treatment facilities have the possibility to participate in this registration to allow a wide coverage of the population.

**Discussion:**

The objective of the paper is to facilitate the use of data by authorities or researchers by correctly describing all aspects of the indicator. The case definition, the variables collected and the way data should be reported are of key importance to use and interpret the data correctly. An overview of the data registered in 2014 gives also an idea of the content of the database. The article also pictures the strengths and limitations of the register and foresees some future improvements.

## Background

Drug policies and action plans require sound and comprehensive evidence on what are the main issues in the drug problematic and how to intervene. In order to obtain a better understanding of the different aspects of the drug phenomenon as well as the impact of related measures, the information exchange, data collection and monitoring of the drug situation at the European level are of great importance [1–3].

Illicit substance use disorders in the general population have a low prevalence. The last Health Interview Survey performed in 2013 in Belgium showed that 2,6 % of the population (15–64 years) is currently (in the last 30 days) using cannabis and 0,8 % has used other illicit substances during the last year [4]. For alcohol, the prevalence is much higher. The Health Interview Survey shows that 10,5 % of the population has a problematic use of alcohol [4]. But such estimations in the general population are often difficult to process due to methodological concerns: representativeness of the sample is often not reached and substance-use disorders are difficult to assess by self-administered questionnaire. Therefore data collected when people with substance use disorders come into contact with health professionals is consequently a main information source for drug epidemiology and demand reduction [5].

The Treatment Demand Indicator (TDI) is an epidemiological indicator collected in a standardised way in all 28 member states of the European Union, Turkey and Norway on behalf of the European Monitoring Centre for Drugs and Drug Addiction (EMCDDA). Through the use of this indicator, insights can be gained into the characteristics, risk behaviours and drug use patterns of people admitted into treatment for their drug use which supports the follow up, ideally in combination with other drug indicators, of trends in the extent and patterns of drug use [5].

### The European protocol

A common protocol for collecting data on people entering drug treatment has been first defined by the Pompidou Group (PG), who coordinated studies at city level (in Dublin and London in 1991) and a developmental project in 11 cities [6]. The PG protocol was published in 1994 [7] and was first implemented at city level and then at country level. In 1994, the EMCDDA was established, and took responsibility for collecting European treatment demand data. The Treatment demand indicator 2.0 protocol [8] was published based on a revision of the first PG protocol. It was preceded by a feasibility assessment concerning methodology and data collection [9] and by an evaluation of national experiences of data reporting using the TDI [10]. Since 2000, the EMCDDA has been implementing the data reporting from the European Union (EU) Member States and adopted formal agreements with the Member States to stimulate and facilitate data collection and reporting from national to European level.

After 10 years of data collection at the European level using this protocol, modification were required so the TDI could better reflect the changes that have occurred over this period not only in the situation of drug use, but also in the treatment system and national and international information systems. Therefore, a third version of the protocol has been set up in 2013 [6]. Today, the indicator is collected in 30 countries (28 member states of the EU, Norway and Turkey) and provides information on almost 400,000 patients in the EU [11].

### The Belgian protocol

Due to organisational problems, Belgium only started the standardization of the TDI data collection in 2006 when all Ministers with a health competencies decided to coordinate the registration of treatment demand and built a specific national protocol based on the European protocol version 2 [12, 13]. Before this time, different initiatives to collect information at different levels (region, city, group of centres,…) on treatment demands of drug use problems took place sometimes since a long time [14–17]. But these registrations were too different to provide a coherent national view of the phenomenon.

Recently, this protocol has been updated in order to include the necessary changes implied by the use of the third European protocol [18]. In the national protocol, the Scientific Institute of Public Health (WIV-ISP) has been appointed as coordinator of the registration and has been asked to develop flexible, secured technical tools to facilitate the data registration in respect with national privacy rules [19–21].

The standardized TDI data collection actually started in Belgium in 2011 based on protocol version 2 and since 2015 based on version 3.

## Methods/Design

### Case definition

In order to better standardize the data collection, a clear case definition is needed and all the terms used need also an explanation in order to avoid confusing interpretation.

The Belgian TDI registration collects information on every treatment episode (d) started by a person (a) in a treatment centre (b) for his/her alcohol or illicit drug use (c).

(a) The registration concerns all individuals without any restriction based on age or nationality. The only condition is that the patient has to have a face-to-face contact with a treatment centre for their substance use problem. Therefore, persons in contact by telephone, letter, internet or contacts which were done by the family of the patient are not included in the registration. Furthermore, every patient has to be informed of the registration for privacy purposes. Especially the existence and objectives of the register, the coordinates of the person responsible for the data, the destination of the data, the right to access and correct their own data have to be mentioned. A patient can resign to participate to this registration by written refusal.

(b) Treatment centres are defined as facilities or practitioners providing treatment for drug or alcohol addiction. These centres offer outpatient or inpatient services, either specialized in addiction treatment or included in larger scale facilities targeting different groups of people. This type of care is sometimes recognized within a convention of authorities such as the national institute for health and disability insurance (NIHDI). The registration of TDI in Belgium is only mandatory for some groups of centres: centres within the NIHDI convention since 2011, centres for mental health in Flanders since 2013, hospitals since 2015, centres with agreement from the Walloon Region since 2011. As a consequence, the number of participating centres varies from year to year and the coverage of the registration can evolve.

Non-professional support groups, centres providing only harm reduction activities, social reintegration, prevention services or outreach activities are not considered as treatment centres.

In order to have an overview of the data collected over time by centres, Table 1 shows the number of registered treatment episodes and the participation rate among all kind of treatment centres potentially be eligible for the TDI registration. These centres are classified in categories describing the type of service as well as the specialized or general aspect of the facility. Coverage in specialized services is much better than in general care settings for both outpatient and inpatient settings. On the other way general practitioners who are playing a major role in detecting and managing substance abuse are not covered by the indicator. Hospitals are increasingly involved in the data collection since 2011 based on pilot projects. From 2015 on a royal decree officialise the data collection in all Belgian hospitals.

**Table 1:** List, participation rate and number of registration episodes of eligible treatment centres in Belgium during the 4 registration years of TDI

	Category	Description	Number of episodes (Participation rate of centres) within a given registration year			
			2011	2012	2013	2014
Outpatient services	Specialized drug treatment centres	Day-care centres	2859 (64 %)	2726 (73 %)	3112 (82 %)	3131 (82 %)
		Consultation centres	416 (31 %)	573 (44 %)	735 (47 %)	1297 (56 %)
		Medical and social care centres	1592 (100 %)	1690 (100 %)	1936 (100 %)	1892 (100 %)
	General/Mental health care	Centres for mental health	24 (1 %)	191 (4 %)	2306 (19 %)	2308 (17 %)
		General practitioners	0 (0 %)	0 (0 %)	0 (0 %)	0 (0 %)
		Psychologists, psychiatrists in private practice	0 (0 %)	0 (0 %)	0 (0 %)	0 (0 %)
		Medical houses	0 (0 %)	0 (0 %)	0 (0 %)	0 (0 %)
	Prisons	Health units in prison	0 (0 %)	0 (0 %)	0 (0 %)	0 (0 %)
Inpatient services	Hospital-based residential drug treatment	Psychiatric hospitals	742 (26 %)	3460 (37 %)	4301 (39 %)	5834 (52 %)
		General hospitals	1055 (10 %)	2966 (22 %)	4171 (24 %)	7381 (38 %)
	Specialized residential treatment (non-hospital based)	Specialized crisis centres	1086 (100 %)	1027 (100 %)	1059 (100 %)	1066 (100 %)
		Therapeutic communities or long-duration residential centre	799 (100 %)	682 (100 %)	727 (100 %)	685 (100 %)
	Supervised housing	Psychiatric care houses	0 (0 %)	0 (0 %)	0 (0 %)	0 (0 %)

(c) Treatment is defined as any activity targeting a person with substance use problems directly in order to obtain results in terms of reducing or eliminating these problems. Possible activities include detoxification or abstinence, substitution treatment, long-term alcohol/drug programmes, psychotherapy, counselling, structured treatment with strong social component, medically assisted treatment, non-medical interventions, specific treatment in prison or interventions aimed at reducing drug-related harm if they are included in a planned programme. Treatment of consequences of substance use in which the use of alcohol or drug is not the main reason for seeking help and sporadic interventions not included in a planned programme are not considered as a treatment. Unlike the European protocol, the Belgian protocol includes alcohol in the list of primary substances. The drug types that are considered : a) opioids (category) including heroin and misuse of methadone, buprenorphine, fentanyl (or illicit) or other opioids; b) cocaine (category) including powder cocaine, crack cocaine or other cocaine; c) stimulants, other than cocaine (category) including amphetamines, methamphetamines, MDMA or derivatives, mephedrone or other stimulants; d) hypnotics and sedatives (category) including misuse of barbiturates and benzodiazepines, GHB/GBL or other hypnotics and misuse of sedatives; e) hallucinogens (category) including LSD, ketamine or other hallucinogens; f) volatile inhalants; g) cannabis (category) including marijuana (herb), hash (resin) or other cannabis and other substances not included above. Tobacco and the use of the substances for a medical treatment or for other somatic or psychiatric reasons are excluded. Non-substance addiction such as gaming or internet addiction is also not part of this registration.

(d) Registration has to be done for every treatment episode defined as the period between the start of the treatment and the end of activities in the context of the program prescribed. The start of the episode is the first face-to-face contact between the professional and the patient. The end of the treatment episode is defined differently in outpatient and inpatient settings. The end of the episode occurs in outpatient settings when the patient stops attending treatment for a period longer than 6 months. In inpatient settings, end of treatment occurs when the patient leaves the centre and no further admission is foreseen. The registration of new treatment episodes is continuous through registration years meaning that a patient with regular visits in an ambulatory setting without a 6 months break, will be registered in TDI only once, namely the first contact with that specific treatment centre.

### Registration system

The WIV-ISP collects and manages data in a secured way at national level and has therefore developed two options for data transfer. The registration module consists of an online form with restricted access for treatment centres in order to encode and manage their data record by record. The repository module is a secured mailbox through which treatment centres can send structured files containing a complete dataset for a given registration year.

The national identification number (NIN) of the patient has to be coded in both options to respect privacy rules before data is stored in the database. The coding is done by a trusted third party (eHealth) and occurs by the run of an algorithm on the field containing the variable for the registration module or on the first specific part of the structured file for the repository module.

In order to better structure the data collection, records from a given year have to be sent by treatment centres before end of March of the next year.

In the next future, this registration system will be integrated into the Healthdata.be process [22] which is a standardized, secured, cross register way of collecting medical data in Belgium. It combines both a database integration and a specific registration tool.

### Collection of the variables

At the start of every treatment episode, a form is completed by a professional during a face-to-face interview with the patient. Questions are related to the identification of the centre, identification of the patient, description of the patient’s socio-demographic and economic status, treatment characteristics and substance use pattern. The questions have slightly changed between the two versions of the Belgian protocol (version 2 (2011–2014) and version 3 (2015-present)). Some questions are also specifically included for centres with a given convention (NIHDI, Hospitals or Walloon Region (WR)). Table 2 summarizes the different variables included in the two versions of the basic form and additional variables corresponding to specific convention centres (Hospitals, NIHDI convention, Walloon Region). A reference to the number of the question is provided in the text below.

**Table 2:** List of variables in the different versions and types of questionnaire of the Belgian TDI protocol

Nr	Variable description	Protocol version 2 (2011–2014)	Protocol version 3 (2015-present)
Identification of the centre			
Q1	Name of the centre	In all versions	In all versions
Q2	Name of the unit/program/satellite	In all versions	In all versions
Q3	Type of unit/program/satellite	In all versions	In all versions
Q4	Type of hospital bed where the patient is hospitalized	/	Version Hospitals
Q5	Arrondissement where the patient is treated	In all versions	In all versions
Q6	Distance between centre and residence place	Version NIHDI	Version NIHDI + Walloon region
Identification of the patient			
Q7	Type of identification	In all versions	In all versions
Q8	Identification number	In all versions	In all versions
Description of the client			
Q9	Sex	In all versions	In all versions
Q10	Age at beginning of the treatment episode	In all versions	In all versions
Q11	Nationality	In all versions	Version NIHDI + Walloon region
Q12	Main type of accommodation during the last 30 days	In all versions	In all versions
Q13	Main type of household during the last 30 days	In all versions	In all versions
Q14	Responsibility of children during the last 30 days	In all versions	In all versions
Q15	Highest educational level achieved	In all versions	In all versions
Q16	Main work situation during the last 30 days	In all versions	In all versions
Q17	Main income situation during the last 30 days	/	In all versions
Description of the treatment			
Q18	Date of the start of treatment	In all versions	In all versions
Q19	Status of previous addiction treatment	In all versions	In all versions
Q20	Source of referral for this treatment episode	In all versions	In all versions
Q21	Lifetime substitution treatment	In all versions	In all versions
Q22	Kind of substitution treatment	In all versions	In all versions
Q23	Age at first substitution treatment	/	In all versions
Q24	Main diagnosis of substance use	Version Hospitals	Version Hospitals
Q25	Treatment objectives	Version Hospitals	Version Hospitals
Substance use patterns			
Q26	Current problematic substances	/	In all versions
Q27	Existence of primary substance	/	In all versions
Q28	Primary substance	In all versions	In all versions
Q29	Other substances used	In all versions	/
Q30	Polydrug use problem	/	In all versions
Q31	Age at first use of primary substance	In all versions	In all versions
Q32	Main route of administration of primary substance	In all versions	In all versions
Q33	Main frequency of use of the primary substance	In all versions	In all versions
Q34	Injecting status	In all versions	In all versions
Q35	Age at first injecting	/	In all versions
Q36	Last injecting occurrence	/	In all versions
Q37	Share of needle status	/	In all versions
Q38	Last share of needle occurrence	/	In all versions
Q39	Share of paraphernalia	/	Version Walloon Region
Q40	Last share of paraphernalia occurrence	/	Version Walloon Region

Identification of the centre is made at the level of the centre itself (Q1) and at the level of a unit or program or satellite within the centre (Q2). The type of treatment unit or program (Q3) and its geographic localisation (Q5) allows to characterize provided treatment and to differentiate geographic areas. The type of hospital bed (Q4) and the distance between the treatment centre and the place of residence (Q6) is also asked in a limited number of centres.

The patient should be identified preferably with the unique NIN (Q8). This number is unique for every Belgian citizen or people having social security rights for non-Belgian citizens. Its use within the TDI registration allows to avoid double counting by identification of the person in different centres at national level and to follow patients between their different treatments episodes. If the NIN of the person is not available or if the patient doesn’t want this number to be registered, it is possible to carry out the registration by mentioning it (Q7).

Variables such as socio-demographic data (sex (Q9), age (Q10) and nationality (Q11)) and socio-economic information (type of accommodation (Q12), type of household (Q13, Q14), educational level (Q15), work and income situation (Q16, Q17)) describe the characteristics of the patient. These variables allow identification of epidemiological groups of patients and assessment of the social relations and stress of the stability of the living situation of the patient.

The start date of the current treatment episode (Q18), the fact that the client already followed previous treatment for substance use before (Q19), the main source of referral (Q20) and his/her substitution treatment situation (Q21, Q22, Q23) are used to describe the treatment situation of the patient and for questions on previous treatment to identify the first treatment entrants. Two questions on diagnosis (Q24) and treatment objectives (Q25) are part of the questionnaire for hospitals.

The variables about the pattern of substance use show important differences between the second and third version of the protocol. In the second version, the primary substance is identified first (Q28) and then the other used substances (Q29). On the contrary, all substances causing problem are identified first (Q26) in version 3 and then eventually (Q27) the primary substance is mentioned (Q28). In this last version, polydrug use (Q30) is identified when more than one substance is causing problem (Q26). Three questions are afterwards related to the patterns of use of the primary substance (Q31, Q32, Q33). Risk behaviour characteristics are then pictured with questions on injecting status (Q34, Q35, Q36) and sharing of syringes (Q37, Q38). In addition, sharing of paraphernalia (Q39, Q40) is asked in some centres.

Table 3 gives a basic view on the content of the database based on some results from the TDI registration in 2014 by type of services. This table shows big differences between the different services in the type of clients seen, their history of treatment and addiction profile.

**Table 3:** General description on the content of the TDI database in 2014 by type of service

	Outpatient services		Inpatient services	
	Specialized drug treatment centres	General/Mental health care	Hospital-based residential drug treatment	Specialized residential treatment (non-hospital based)
Proportion of use of the national identification number	70 %	60 %	66 %	73 %
Proportion of women	19 %	26 %	32 %	18 %
Mean age	32	NA	43	33
Top 3 problematic substances	1. Cannabis (31 %)	1. Alcohol (51 %)	1. Alcohol (72 %)	1. Opiates (33 %)
	2. Opiates (29 %)	2. Cannabis (27 %)	2. Opiates (7 %)	2. Cocaine (20 %)
	3. Cocaine (14 %)	3. Cocaine (6 %)	3. Cannabis (7 %)	3. Alcohol (18 %)
Proportion first treatment ever	44 %	54 %	24 %	13 %
Top 3 sources of referral	1. Self-referred (40 %)	1. Justice (25 %)	1. Self-referred (47 %)	1. Self-referred (46 %)
	2. Justice (22 %)	2. Self-referred (19 %)	2. Hospital (14 %)	2. Addiction centre (24 %)
	3. Other (16 %)	3. Hospital (17 %)	3. Other (14 %)	3. Hospital (12 %)

### Analysis and reporting

Quality checks are performed directly in the registration module or after reception of the file from the repository module. Checks are related to the content of the variable (values in every variable), and to compatibility between variables (the value in one variable is dependent on the value in others). No quality checks are currently made on a longitudinal basis (comparing data of the same patient in time) but it will be possible to implement this in the future.

Data is stored in a datawarehouse where each record correspond to a treatment episode. Each treatment episode is identified by the coded identification number of the patient, identification number of the treatment unit and the start date of treatment.

For those registered with the NIN it’s possible to identify double records being the same patient registered in the same unit at the same start date of treatment. It’s also possible to identify treatment episodes for the same person over time.

Data from the database can be used either as the number of treatment episodes or the number of patients. The number of patients however is an overestimation as all anonymously recorded registrations are counted as separate patients.

Data providers have access to their raw data registered in the database in order to allow them to check and perform analysis on their own data. In addition a reporting module has been developed to present the data in tables and figures, comparing the descriptive statistics with results of other similar centres. Some authorities also receive raw data concerning treatment centres under their responsibility. As the collection of the indicator info is commissioned by the EMCDDA, an obligatory report is sent annually to the EMCDDA in aggregated tables. Reports on specialized centres with NIHDI convention have been written annually since 2011 [23–26].

A steering committee (CocoTDI), including representatives of treatment centres, authorities and national experts is appointed to follow-up the registration and provides advices on the use and registration of data.

### Use of TDI data

TDI data must be used for epidemiological purposes only. The use of these data in order to assess administrative objectives is not possible. Data is well adapted to describe characteristics of groups of patients starting a treatment for substance use, to assess patterns of use, to follow trends in time and to describe the use of services by patients. In combination with other databases, further in-depth analyses will also be possible.

The use of NIN makes also possible the linkage between different databases. A recent study proposed to link the TDI database with the health insurance databases [27].

At European level, the TDI data makes comparisons between countries possible for a given phenomenon like the recent study on heroin use and heroin injection [28] or the study on trends of injecting drug use [29].

The interpretation of TDI data should be made with caution as data concern a very specific population. The use of absolute numbers should always consider the coverage of the data collection and only similar data can be compared. The terminology used to interpret and clarify the data should always be chosen carefully. For example, it’s sometimes easier to speak more generally about drug user whereas TDI data is only about those starting a treatment. Furthermore the clients registered in TDI cannot be automatically considered as problematic users according to the most common definitions.

A website is dedicated to the technical aspects of the TDI registration in Belgium (https://tdi.wiv-isp.be) and provides also a list of publications based on TDI data. From data collection 2015 on, a national report will present the detailed situation of the treatment demand in Belgium. A procedure is going to be approved in order to standardize and clearly document the requests for data from the TDI database for specific research.

## Discussion

The TDI database is a valuable epidemiological database as it represents one of the rare reliable data pools available on drug users in treatment. Its national and European range and its longitudinal objective make it also an interesting tool to compare between European countries, subgroups of patients, specific geographic areas and over time.

Every country has to adapt the European protocol to its own specific requirements and possibilities. The Belgian TDI protocol is the result of a consensus with national experts and authorities, meeting as much as possible the European standards. In order to safeguard some national specificities, variants of the questionnaire are existing to target specific questions for a limited number of centres. Nevertheless some variables from the European version have not been considered in the Belgian version (such as questions on testing for infectious diseases), while some items have been differently implemented (such as polydrug use). Also other items like for example alcohol as primary substance are integrated.

The TDI protocol has been officially approved by all ministers of public health and published in the official gazette which gives an official status and a clear visibility for all actors concerned. The approvals from the privacy commission and its future integration in a database system of Healthdata.be also offers a token of a correct way of dealing with personal data.

The strength of this registration is the use of the NIN as unique identification number of clients as this increases the utility of these data. In addition the identification of double records and treatment episodes of a client in centres at national level, this permits to follow up of a client’s pathway through treatment facilities and the evolution of their patterns of use in time. The possibility to link these data with other databases taken the privacy rules into account at any time, creates the possibility to use this indicator for in-depth scientific analyses. However the registration of the national identification number is not mandatory. This allows to register data even from patients not having a NIN nor willing to share it. In consequence, not all potential duplicates can be excluded and about 30 % of the data cannot be linked to other treatment episodes within the TDI register nor to other databases.

Even though the representativeness of demand in specialized centres and in general/psychiatric hospitals is relatively good, TDI is facing a lack of registration in the non-specialized sector (medical house, centres for mental health, private practice,…). Consequently, the tool offers a valuable information about drugs or alcohol users reached by specialized care centres and general/psychiatric hospitals but the profile of patients may not currently be generalised to the whole patients seeking help for drug use as we observe great differences in the patient’s characteristics among the sectors. Enhance the representativeness of the general sector is one of the main action to tackle in the future.

More knowledge on the coverage aspects of the indicator is needed to better evaluate the validity of the available information. Coverage can be interpreted in two ways: as “external coverage” being the number of centres reporting compared to the total number of centres in the country as well as “internal coverage” being the number of individuals and of treatment episodes registered compared with the total number of treatment episodes started. This aspect could be targeted in the future through the use of a regular treatment facility survey which is in development.

The TDI registration is sometimes criticized for having a definition of ‘treatment’ or ‘treatment centre’ that is too limited. Contacts in harm reduction settings (syringe exchange programmes) or treatment of complications of drug use are not registered. From an international perspective, further development of the TDI indicator is always possible and if a consensus can be reached, the protocol could be reviewed in order to better assess these issues in a structured and coordinated way.

The present TDI indicator reflects the incidence of new started treatments for a drug and/or alcohol problem. On the contrary the treatment prevalence, being the total number of clients in treatment (including also long-term treated clients), cannot be assessed with the present TDI indicator. The prevalence is interesting for assessment of actual workload in treatment services. Such a new indicator could relatively easily be combined with the TDI registration again based on the use of the NIN.

TDI in Belgium has been implemented since 2011 and is now almost functioning in a routine way. This is the first time a national overview is approached that gives information on the demands for substance related treatment. The use of the data should be supported in the future to better demonstrate its relevance in health care research and policy making and to promote the data registration at the level of field organizations with focus on quantity as well as on quality.

## Abbreviations

EMCDDA, European Monitoring Center for Drug and Drug Addiction; EU, European Union; NIN, National Identification Number; NIHDI, National Institute for Health and Disability Insurance; PG, Pompidou Group; TDI, treatment demand indicator.
